# Beluga whale pVHL enhances HIF-2α activity via inducing HIF-2α proteasomal degradation under hypoxia

**DOI:** 10.18632/oncotarget.15038

**Published:** 2017-02-02

**Authors:** Jianling Bi, Bo Hu, Jing Wang, Xing Liu, Jinsong Zheng, Ding Wang, Wuhan Xiao

**Affiliations:** ^1^ The Key Laboratory of Aquatic Biodiversity and Conservation, Institute of Hydrobiology, Chinese Academy of Sciences, Wuhan, 430072, P. R. China; ^2^ State Key Laboratory of Freshwater Ecology and Biotechnology, Institute of Hydrobiology, Chinese Academy of Sciences, Wuhan, 430072, P. R. China

**Keywords:** beluga whale, cetaceans, hypoxia, HIF-2α, VHL

## Abstract

Aquatic mammals, such as cetaceans experience various depths, with accordingly diverse oxygenation, thus, cetaceans have developed adaptations for hypoxia, but mechanisms underlying this tolerance to low oxygen are unclear. Here we analyzed VHL and HIF-2α, in the hypoxia signaling pathway. Variations in VHL are greater than HIF-2α between cetaceans and terrestrial mammals, and beluga whale VHL (BW-VHL) promotes HIF-2α degradation under hypoxia. BW-VHL catalyzes BW-HIF-2α to form K48-linked poly-ubiquitin chains mainly at the lysine 429 of BW-HIF-2α (K429) and induces BW-HIF-2α for proteasomal degradation. W100 within BW-VHL is a key site for BW-VHL functionally and BW-VHL enhances transcriptional activity of BW-HIF-2α under hypoxia. Our data therefore reveal that BW-VHL has a unique function that may contribute to hypoxic adaptation.

## INTRODUCTION

Cetaceans (whales, dolphins, and porpoises) are aquatic mammals that must dive for food and likewise must adapt to a range of oxygen and temperature changes [1, 2]. The sperm whale (*Physeter microcephalus*) can dive deeper than 2000 m and remain submerged more than 2h, whereas the Yangtze finless porpoise (*Neophocaena asiaeorientalis*) can dive to within 20 m and stay under water less than 2 min [3, 4]. Similar to that of the Yangtze finless porpoise, the Baiji (the Yangtze River dolphin) (*Lipotes vexillifer*), which also lived in the Yangtze river, can only dive to the depth shorter than 20 m and stay submerged less than 5 min [5–7]. The beluga whale (*Delphinapterus leucas*), chiefly an Arctic Ocean inhabitant [8], can dive to 650 m and stay under water 25 min [9, 10]. But, compared to the sperm whale which resides in warmer water, the beluga whale habitat is relatively narrow and includes very low temperature [11], that may include ice and hypoxia [12, 13].

Oxygen, a final electron acceptor for the generation of ATP, controls developmental processes and tissue homeostasis [14]. Thus, most terrestrial mammals are sensitive to hypoxia and apnea for 3-4 min can be fatal; hypoxia for 15-20 sec in human can cause loss of consciousness [15]. Mammals, unlike fish, must absorb oxygen via respiring air on the water surface, but for predation, cetaceans must dive to various depths for different durations [2, 16, 17].

To adapt to this diving need, cetaceans have an acute hypoxia tolerance arising from breath holding during diving [18], as well as innate mechanisms for attenuating damage from reactive oxygen stress (ROS) due to re-oxygenation during the return to the water surface [19–21]. Compared to hypoxic tolerance in terrestrial mammals, such as plateau and cave mammals which adapt to chronic hypoxia, cetaceans have adapted to fluctuating oxygen [22, 23], but how this occurs is unclear [24, 25].

To date, the hypoxia adaptation in mammals has been studied in plateau mammals (including humans) and cave mammals [26–28] and data show that for oxygen homeostasis, mammals use a hypoxia signaling pathway to facilitate oxygen delivery and cellular adaptation to oxygen deprivation [29]. Multiple lines of evidence indicate that the hypoxic adaptation and tolerance of mammals is governed by the hypoxia signaling pathway [27] [30–33], in which, hypoxia-inducible factor 1α (HIF-1α) and 2α (HIF-2α) are two master regulators that coordinate adaptive cellular response to hypoxia by activating gene expression to control metabolism, glucose uptake, erythropoiesis, angiogenesis and cell survival [29].

Under normoxia, prolyl hydroxylase enzymes (PHD1-3) use oxygen as a substrate to hydroxylate key proline residues within HIF-1α and HIF-2α, allowing von Hippel-Lindau protein (pVHL), the substrate recognition component of an E3 ubiquitin ligase complex, to bind to hydroxylated HIF-1α and HIF-2α and target them for proteasomal degradation [34–37]. Under hypoxia, PHD activity is inhibited and pVHL can not binding to HIF-α, resulting in HIF-α stabilization and nuclear translocation where HIF-α subunits dimerize with aryl hydrocarbon receptor nuclear translocator (ARNT) and bind to hypoxia-responsive elements in target genes to activate gene transcription [38, 39]. Therefore, the regulation of HIF-α stability by pVHL is considered to be a key step for controlling the hypoxia signaling pathway [12]. Defining mechanisms of regulation of HIF-α by pVHL will increase our understanding of the hypoxia signaling pathway as well as help us to understand the mechanisms of hypoxic adaptation and tolerance.

Previously, we characterized behaviors and functions of key factors of the hypoxia signaling pathway in cetaceans, such as HIF-1α from the sperm and beluga whale and the Yangtze finless porpoise [40]. Here, we characterized HIF-2α and its regulation by pVHL and we noted that beluga whale pVHL enhanced HIF-2α activity by inducing HIF-2α proteasomal degradation under hypoxia, which is different from pVHL functions described to date [12, 41]. This finding may explain the beluga habitat and assist with understanding evolutionary biology and functional adaptation.

## RESULTS

### BW-VHL promotes BW- HIF-2α turnover under hypoxic and normoxic conditions

**Figure 1:**
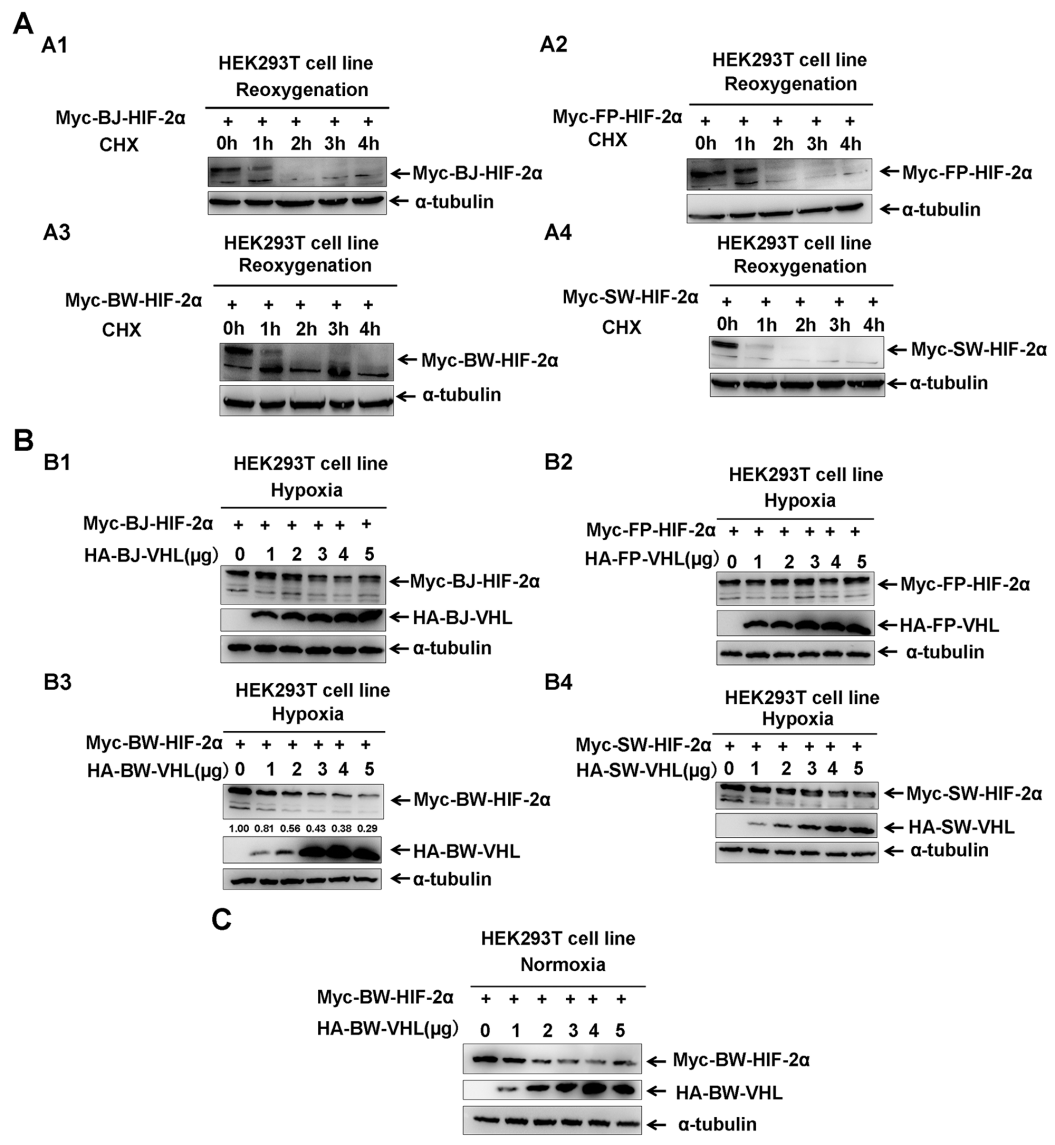
The beluga whale (BW) VHL promotes the beluga whale HIF-2α turnover under both hypoxic and normoxic conditions **A**. HIF-2α was degraded in response to reoxygenation. **A1**. The Baji (BJ) HIF-2α; **A2**. the Yangtze finless porpoise (FP) HIF-2α; **A3**. the beluga whale (BW) HIF-2α; **A4**. the sperm whale (SW) HIF-2α. HEK293T cells were transfected with Myc tagged HIF-2α expression vectors, then incubated in hypoxic conditions (1 % O2) for 18–20 h followed by normoxia (21 % O2) in the presence of protein synthesis inhibitor cycloheximide (CHX, 50μg/mL); the proteins levels of HIF-2αfrom the baiji, the Yangtze finless porpoise, the beluga whale, and the sperm whale were detected by Western blot analysis at different time points after exposure to normoxia. **B**. The beluga whale VHL promotes the beluga whale HIF-2α turnover under hypoxia, but VHL from the baiji, the Yangtze finless porpoise and the sperm whale had no obvious effect on the protein stability of their HIF-2α. **B1**. The Baji (BJ) HIF-2α and VHL; **B2**. the Yangtze finless porpoise (FP) HIF-2α and VHL; **B3**. the beluga whale (BW) HIF-2α and VHL; **B4**. the sperm whale (SW) HIF-2α and VHL. HEK293T cells were transfected with equal amounts of Myc-HIF-2α along with increasing amounts of HA-VHL, compensated with a CMV-HA empty vector to keep the same amount of transfected plasmid DNA. The protein levels of HIF-2α from the baiji, the Yangtze finless porpoise, the beluga whale, and the sperm whale were detected by Western blot analysis. **C**. The beluga whale VHL promoted the beluga whale HIF-2α degradation under normoxia in a dose-dependent manner.

Similar to HIF-1α, HIF-2α is evolutionarily conserved in cetaceans (Supplementary Figure 1). In response to reoxygenation, HIF-2α from the Baji (BJ), Yangtze finless porpoise (FP), beluga whale (BW), and sperm whale (SW) degraded quickly (Figure 1A), which is a typical feature of HIF-2α protein [29]. However, comparing pVHL sequences among these four cetaceans and humans, pVHL was not as evolutionarily conserved as HIF-2α, particularly in the N-terminal of pVHL (Supplementary Figure 2). Thus, we next studied whether cetaceans’ pVHL is unique regarding the aquatic environment compared to the terrestrial habitat. Similar to human pVHL, four cetacean pVHLs, including BJ-VHL, FP-VHL, BW-VHL and SW-VHL could induce their HIF-2α degradation under normoxic conditions (data not shown). However, under hypoxia, BW-VHL could induce BW-HIF-2α degradation in a dose-dependent manner, but the other three VHL (BJ-VHL, FP-VHL and SW-VHL) did not change HIF-2α stability (Figure 1B).

**Figure 2:**
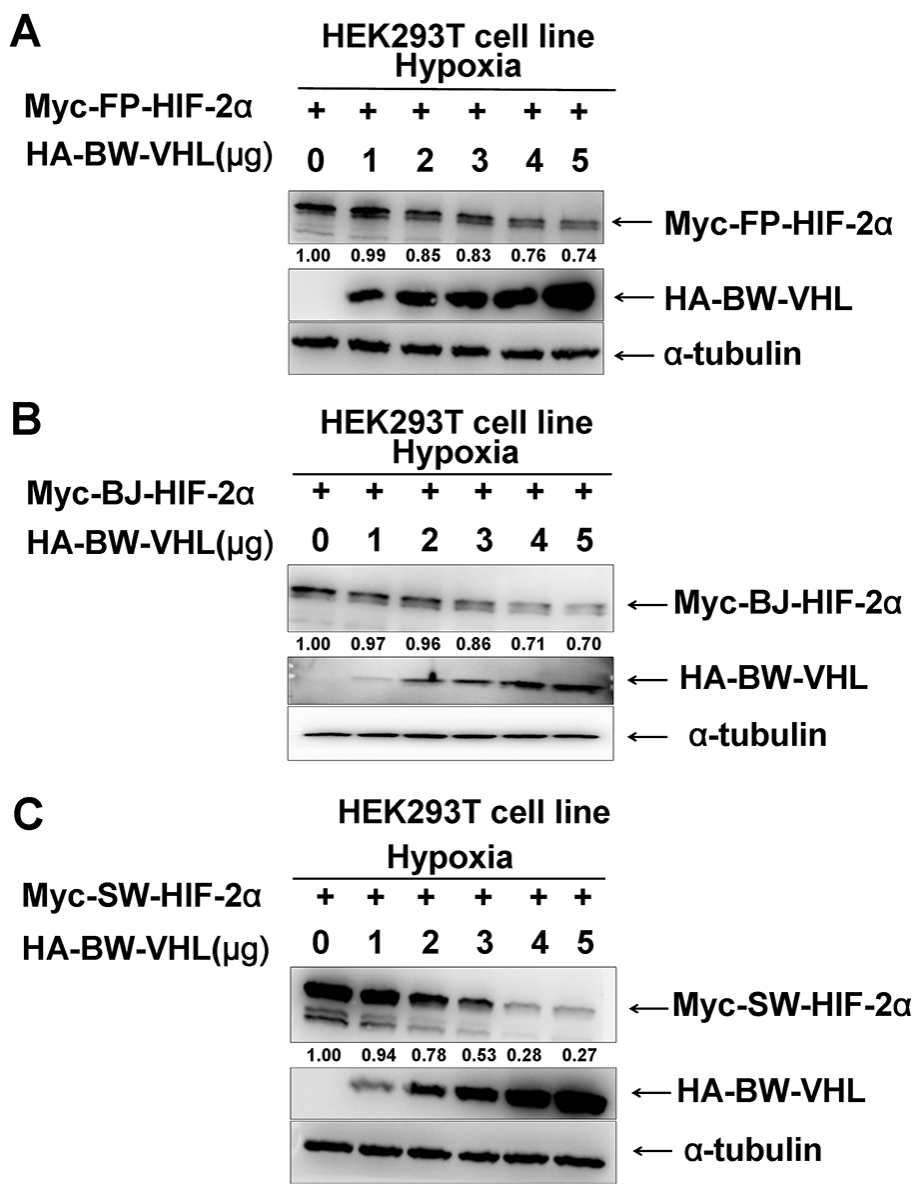
The beluga whale (BW) VHL promotes the degradation of HIF-2α from the Yangtze finless porpoise (FP), the baiji (BJ) and the sperm whale (SW) under hypoxia **A**. Yangtze finless porpoise (FP) HIF-2α; **B**. the Baji (BJ) HIF-2α; **C**. the sperm whale (SW) HIF-2α. HEK293T cells were co-transfected with equal amounts of HIF-2α from the Yangtze finless porpoise, the baiji and the sperm whale along with increasing amounts of expression vector encoding beluga whale VHL.

BW-VHL activity was different from that reported for pVHL [12, 42]. BW-HIF-2α degradation by BW-VHL under normoxia was confirmed (Figure 1C), suggesting that BW-VHL may uniquely modulate hypoxia signaling. To explore the mechanisms underling this process, we attempted to exclude BW-VHL-mediated BW-HIF-2α degradation being due to BW-HIF-2α by investigating the effect of BW-VHL on FP- HIF-2α, BJ- HIF-2α and SW- HIF-2α under hypoxia. Figure 2 shows that BW-VHL caused degradation on all HIF-2α forms, so, BW-VHL can induce HIF-2α degradation under hypoxia.

**Figure 3:**
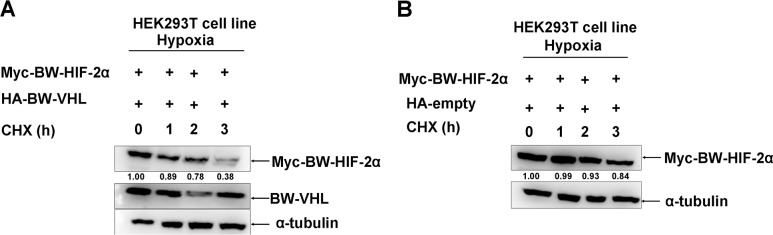
The beluga whale VHL promotes the beluga whale HIF-2α degradation in the presence of CHX under hypoxia **A**. Myc tagged BW-HIF-2α was co-transfected with HA tagged BW-VHL. **B**. Myc tagged BW-HIF-2α was co-transfected with HA empty vector. HEK293T cells were transiently transfected with the indicated plasmids then incubated under hypoxia. After 24 h, cycloheximide (CHX) was added to the cells under hypoxia condition, and cell lysates were prepared at the indicated time points.

Subsequently, we used cyclohexamide to block new protein synthesis and examined BW-HIF-2α stability when BW-VHL was overexpressed under hypoxia. Figure 3 shows that compared to co-transfection with empty vector control, co-transfection with BW-VHL caused BW-HIF-2α degradation, suggesting that no new protein synthesis was required for BW-VHL-inducing HIF-2α degradation.

**Figure 4:**
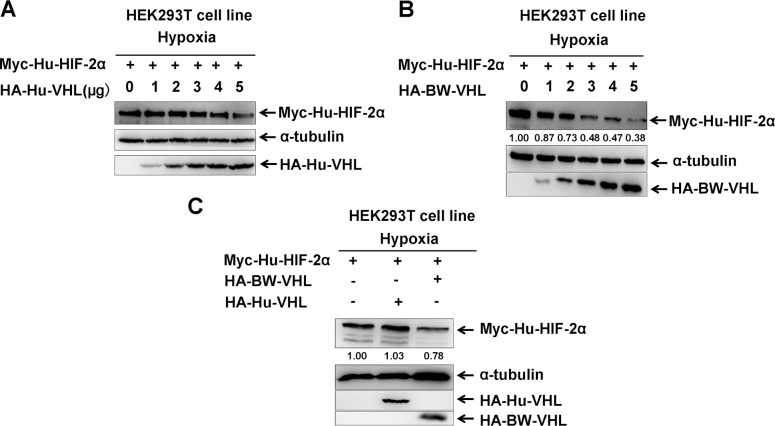
The beluga whale VHL promotes the degradation of human HIF-2α under hypoxia **A**. Human VHL had no obvious effect on human HIF-2α protein level under hypoxia. **B**. Overexpression of the beluga whale VHL in HEK293T cells promotes human HIF-2α degradation in a dose-dependent manner under hypoxia. **C**. The beluga whale VHL promoted human HIF-2α degradation, but human VHL did not do so.

**Figure 5:**
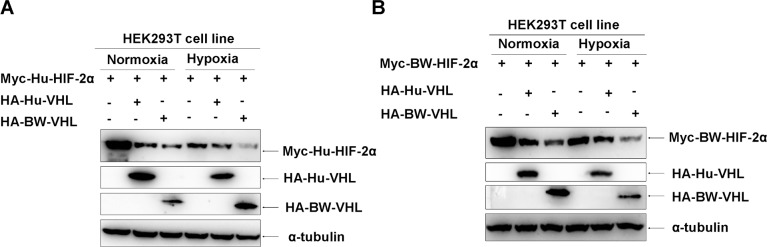
The beluga whale (BW) VHL induces degradation of human and beluga whale HIF-2α under hypoxia, but human VHL does not do so **A**. Under normoxia, both the beluga whale VHL and human VHL induced degradation of human HIF-2α; but under hypoxia, only the beluga whale VHL induced degradation of human HIF-2α and human VHL did not do so. **B**. Under normoxia, both the beluga whale VHL and human VHL induced degradation of the beluga whale HIF-2α; but under hypoxia, only the beluga whale VHL induced degradation of the beluga whale HIF-2α and human VHL did not do so.

Due to lack of suitable cetacean cell lines, human cell lines were used and potential gene evolutionary conservation of gene functions between cetaceans and humans were kept in mind. Studies to determine whether BW-VHL could induce human HIF-2α degradation under hypoxia in human HEK293T cell line suggested that human VHL (Hu-VHL) could not induce human HIF-2α degradation (Figure 4A), but BW-VHL could indeed induce Hu-HIF-2α degradation under hypoxia (Figure 4B and 4C). In addition, under normoxia, both Hu-VHL and BW-VHL could induce Hu-HIF-2α and BW-HIF-2α degradation (Figure 5), similar to pVHL from other mammals [31, 34]. With hypoxia, only BW-VHL could induce both Hu-HIF-2α and HIF-2α degradation (Figure 5). These data not only indicate that induction of HIF-2α degradation under hypoxia due to BW-VHL is unique, but also indicates that human cell lines are feasible for assaying hypoxia signaling pathways of cetaceans.

**Figure 6:**
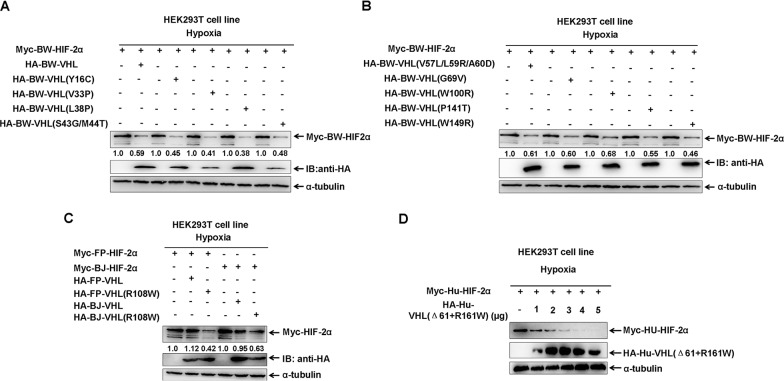
The effect of beluga whale (BW) VHL mutants on HIF-2α protein stability under hypoxia **A-B**. Comparisons of different VHL mutants on the stability of beluga whale HIF-2α. The ability of beluga whale VHL mutant (W100R) on promoting HIF-2α degradation was the lowest among the different VHL mutants. **C**. The Baji (BJ) VHL mutant (R108W) induced the Baji (BJ) HIF-2α degradation, and the Yangtze finless porpoise (FP) VHL mutant (R108W) induced the Yangtze finless porpoise (FP) HIF-2α degradation. **D**. Human VHL mutant (Δ61+R161W) induced human HIF-2α degradation. HEK293T cells were co-transfected with equal amounts of human HIF-2α with increasing amounts of human VHL mutant (Δ61+161R/W) under hypoxia.

**Figure 7:**
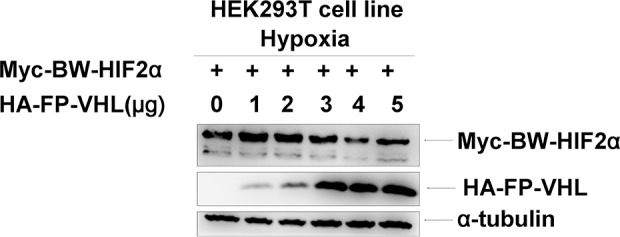
The Yangtze finless porpoise (FP) VHL has no obvious effect on the stability of the beluga whale (BW) HIF-2α under hypoxia HEK293T cells were co-transfected with equal amounts of Myc-tagged BW-HIF-2α expression vector along with increasing amounts of HA-tagged FP-VHL expression vector under hypoxia.

To elucidate why BW-VHL could induce HIF-2α degradation, but Hu-VHL could not, we compared BW-VHL and Hu-VHL sequences: BW-VHL lacked the 61 amino-acids of the N-terminal of Hu-VHL (Supplementary Figure 3). In addition, about 20 amino acids were different between BW-VHL and Hu-VHL, excluding the 61 amino acids at N-terminal (Supplementary Figure 3). To determine whether BW-VHL was unique due to these mutations, we mutated 12 residues of BW-VHL, including Y16, V33, L38, S43, M44, V57, L59, A60, G69, W100, P141 and W149, to mimic amino acids of Hu-VHL. Data show that (Figure 6A and 6B) all mutants could induce BW-HIF-2α degradation to a various degree. Also, among these mutants of BW-VHL, BW-VHL(W100R) had the weakest ability to induce BW-HIF-2α. Of note, W100 of BW-VHL corresponded to R161 of human VHL. Thus, we next studied whether R mutated to W at site 100 of BW-VHL might explain its capability to induce HIF-2α under hypoxia. Assays indicate that BJ-VHL and FP-VHL lacked the N-terminal (53 amino acids) of Hu-VHL, but they have R in a position corresponding to W100 of BW-VHL, and R161 of Hu-VHL. In addition, FP-VHL could not effectively induce FP-HIF-2α and BW-HIF-2α degradation (Figure 1B2; Figure 7) and BJ-VHL could not effectively induce BJ-HIF-2α degradation (Figure 1B1) under hypoxia. Thus, lack of a N-terminus of BW-VHL might not explain induction of HIF-2α degradation under hypoxia.

To further determine whether W100 in BW-VHL explains its function, we mutated the conserved R residue of BJ-VHL to W, as well as that in FP-VHL: FP-VHL(R108W) and BJ-VHL(R108W). Similar to BW-VHL, FP-VHL(R108W) and BJ-VHL(R108W) could induce their own HIF-2α degradation under hypoxia, but wild-type VHL, FP-VHL and BJ-VHL, did not do so (Figure 6C).

To match the structure and sequence of wild-type BW-VHL, we deleted the first 61 amino acids of Hu-VHL's N-terminal and mutated its R161 to W (Hu-VHL(Δ61+R161W)). The mutant, Hu-VHL(Δ61+R161W), could also induce Hu-HIF-2α degradation under hypoxia (Figure 6D). Therefore, BW-VHL with W100 may explain its ability to induce HIF-2α degradation under hypoxia.

**Figure 8:**
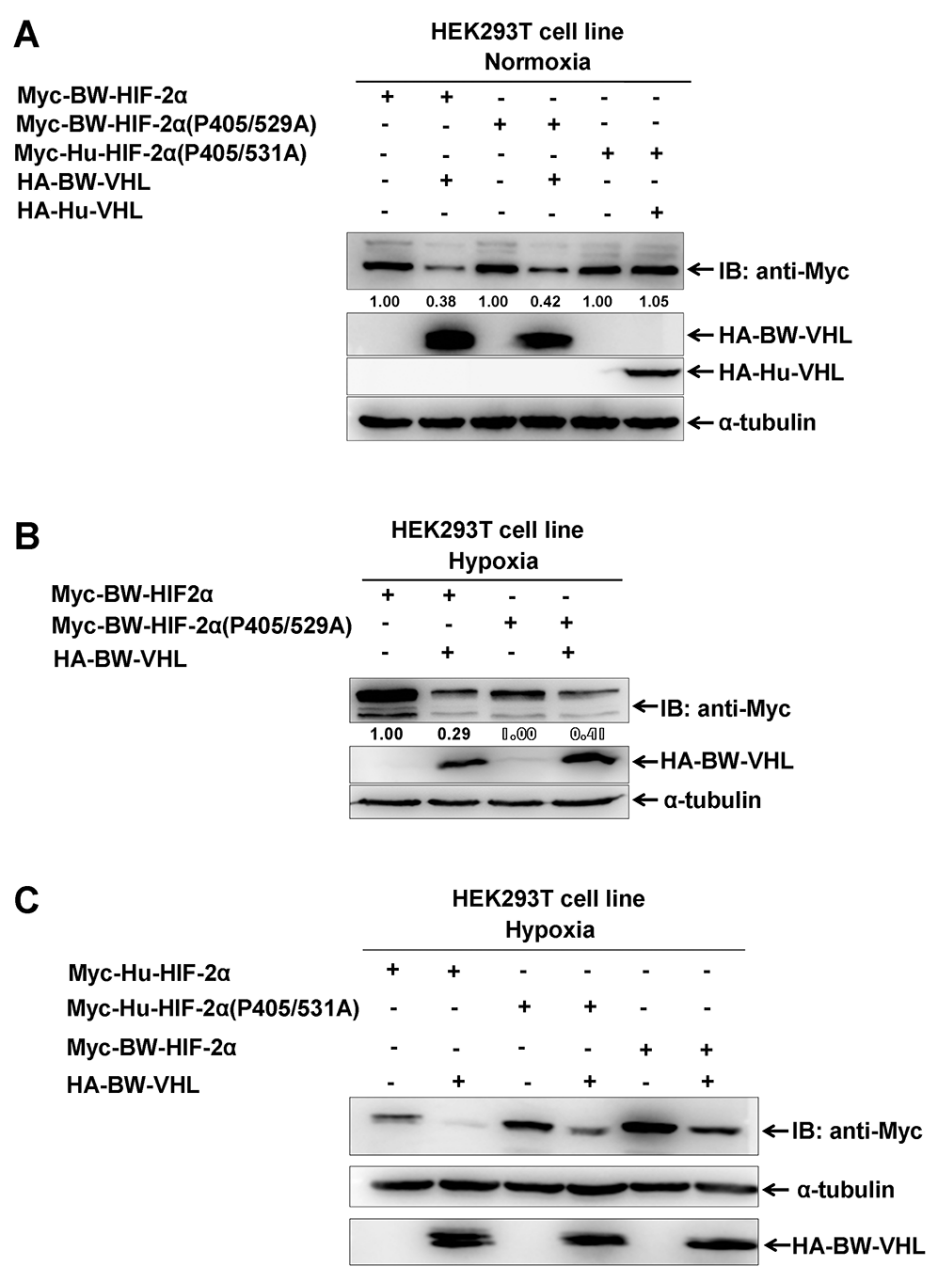
The degradation of HIF-2α by beluga whale VHL is independent of prolyl hydroxylation **A**. Overexpression of the beluga whale VHL leaded to degradation of both the wildtype beluga whale HIF-2α and the potential hydroxylation proline-site-mutated beluga whale HIF-2(P405/529A) under normoxia; but overexpression of human VHL did not induce degradation of the hydroxylation proline-site-mutated human HIF-2(P405/531A) under normoxia. **B**. Overexpression of the beluga whale VHL leaded to degradation of both the wildtype beluga whale HIF-2α and the potential hydroxylation proline-site-mutated beluga whale HIF-2(P405/529A) under hypoxia. **C**. Overexpression of the beluga whale VHL leaded to degradation of both the wildtype human HIF-2α and the hydroxylation proline-site-mutated human HIF-2(P405/531A) under hypoxia.

To determine whether HIF-2α degraded by BW-VHL under hypoxia depends on proline hydroxylation, we created a BW-HIF-2α mutant, in which two proline sites (P405 and P529), corresponding to P405 and P531 of human HIF-2α, were mutated to alanine (A). Under normoxia, BW-VHL could induce the proline site mutated mutantion of BW-HIF-2α (BW-HIF-2α(P405/529/A)) degradation, and wildtype BW-HIF-2α. However, Hu-VHL could not induce proline-site mutated mutant of Hu-HIF-2α degradation as reported (Figure 8A) [29]. Under hypoxia, BW-VHL could induce both wild-type and the proline-site mutated mutant of BW-HIF-2α degradation (Figure 8B). With hypoxia, BW-VHL could induce both wild-type and the proline-site mutated mutant of Hu-HIF-2α degradation as well (Figure 8C). Therefore, BW-VHL mediated HIF-2α degradation under both normoxia and hypoxia is independent of proline hydroxylation.

### BW-VHL catalyzes poly-ubiquitination and induces proteasomal degradation of BW-HIF-2α under hypoxia

**Figure 9:**
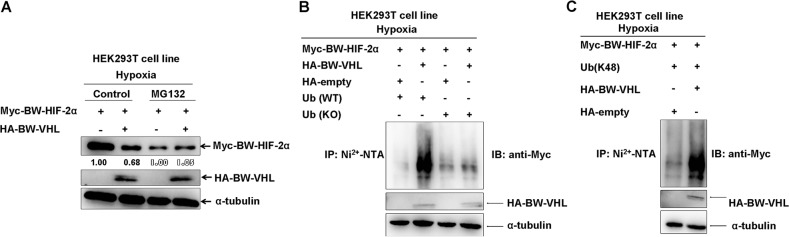
The beluga whale (BW) VHL catalyzes the beluga whale HIF-2α to form K48-linked ubiquitination, resulting in proteasomal degradation under hypoxia **A**. The proteasome inhibitor MG132 could block the beluga whale VHL-induced the beluga whale HIF-2α degradation under hypoxia. HEK293T cells were transfected with Myc-tagged BW- HIF-2α together with either HA-tagged BW-VHL or empty vector under hypoxia; MG132 (20 μM) was added to the medium 6 h before protein harvest. **B**. The beluga whale (BW) VHL enhanced poly-ubiquitination of BW-HIF-2α under hypoxia but it did not induce poly-ubiquitination of BW-HIF-2α in the presence of lysine-null ubiquitin (KO). HEK293T cells were transfected with His-tagged Ub or Ub (KO), Myc-tagged BW-HIF-2α along with HA empty vector, or HA-tagged BW-VHL. After transfected for 24 hours, lysates were subjected to immunoprecipitation by Ni2^+^- NTA beads, then were detected by Western blot analysis using anti-Myc antibody. **C**. The beluga whale VHL catalyzed the beluga whale HIF-2α to form K48-linked ubiquitination under hypoxia. HEK293T cells were transfected with Myc-tagged BW-HIF-2α and HA-tagged BW-VHL, with His-tagged ubiquitin mutant (Ub-K48 only), the ubiquitination assays were performed as that described as that of **B**.

Studying whether BW-VHL induced BW-HIF-2α proteasomal degradation, we noted that (Figure 9A), adding of MG132 blocked BW-HIF-2α degradation induced by BW-VHL under hypoxia (right two panels in Figure 9A). BW-VHL could catalyze poly-ubiquitin chain formation of BW-HIF-2α in the presence of wild-type ubiquitin (WT), but not of lysine mutated ubiquitin (KO) under hypoxia (Figure 4B). Furthermore, BW-VHL catalyzed BW-HIF-2α to form K48-conjugated poly-ubiquitin chains under hypoxia, consistent with its role in inducing proteasomal degradation (Figure 9C).

Because VHL is a component of the VBC E3 ubiquitin ligase complex and E3 ubiquitin ligase usually catalyze its targets at lysine (K) residue (s) to form poly-ubiquitin chains, we next determined which lysine residue(s) in BW- HIF-2α was (were) catalyzed by BW-VHL. BW-VHL promotes BW-HIF-2α degradation under hypoxia, so we used protein degradation efficiency by BW-VHL to monitor the potential ubiquitination site(s) in BW-HIF-2α. Pilot experiments indicated that BW-VHL could not induce BW-HIF-1α degradation under hypoxia (Data not shown), and the eleven lysine sites (K150, K349, K385, K429, K585, K592, K675, K680, K700, K702 and K736) which were not evolutionarily conserved between BW- HIF-1α and BW-HIF-2α, but were evolutionarily conserved among cetaceans, human, mouse and rat, were chosen to mutate to arginine (R).

**Figure 10:**
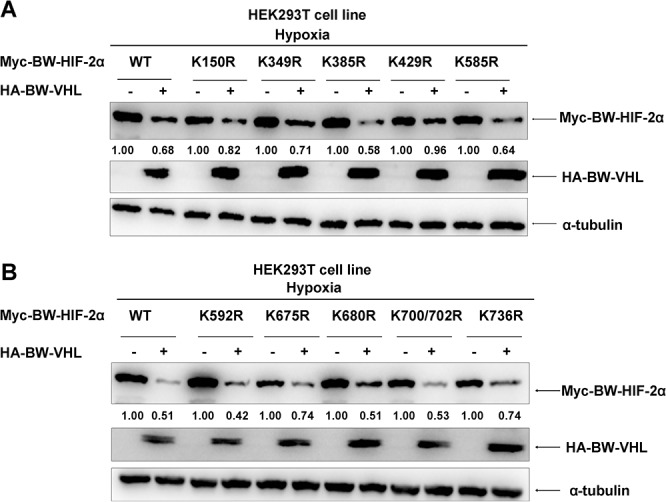
The effect of beluga whale VHL on the stability of HIF-2α mutants **A-B**. Comparisons of the effect of the beluga whale VHL on stability of HIF-2α mutants. Among HIF-2α mutants, HIF-2α (K429R) was the most resistant for BW-VHL-induced degradation. HEK293T cells were transfected with the indicated beluga whale HIF-2α mutants together with either beluga whale VHL or empty vector under hypoxia. The expressions of beluga whale HIF-2α were detected by Western blot analysis.

**Figure 11:**
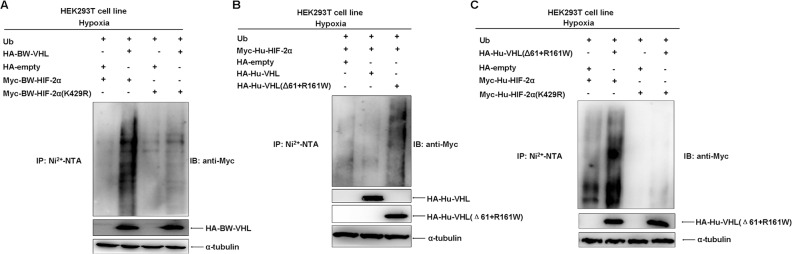
The beluga whale VHL targets the beluga whale HIF-2α for poly-ubiquitination at K429 under hypoxia **A**. The catalytic capability of the beluga whale VHL on the beluga whale HIF-2α (K429R) polyubiquitination is reduced obviously compared to that on wild-type BW-HIF-2α under hypoxia. HEK293T cells were transfected with Myc-tagged BW-HIF-2α or BW-HIF-2α mutant (K429R) along with HA-tagged BW-VHL and His-tagged ubiquitin, the ubiquitination assays were conducted. **B**. Human VHL mutant (Δ61+161R/W) significantly enhanced the poly-ubiquitination of human HIF-2α under hypoxia. **C**. The catalytic capability of human VHL mutant (Δ61+161R/W) on human HIF-2α mutant(K429R) polyubiquitination was reduced significantly compared to that on wild-type human HIF-2α under hypoxia.

Overexpression of BW-VHL promoted degradation of virtually all mutants to a various degree under hypoxia (Figure 10A and 10B). However, BW-VHL induced K429R mutant degradation the least (1.00 versus 0.96; Figure 10A). Thus, K429 in BW-HIF-2α may be the key site catalyzed by BW-VHL to form poly-ubiquitin chains under hypoxia. To test this, we used ubiquitination assays and data (Figure 11A) show that BW-VHL could catalyze wild-type BW-HIF-2α to form polyubiquitin chains, but not of BW-HIF-2α(K429) mutant.

To know whether this feature of BW-VHL is specific, we tested it with human VHL and human HIF-2α. Human wild-type VHL (Hu-VHL) could not catalyze human HIF-2α (Hu-HIF-2α) to form polyubiquitin chains, but human VHL mutant (Δ61+R161W) could do so under hypoxia, suggesting that this feature of BW-VHL is really unique and may be used to control the stability of HIF-2α from other species (Figure 11B). To better understand this, we validated the effect of the mutant, Hu-VHL (Δ61+R161W) on human wild-type HIF-2α and the mutant HIF-α (Hu-HIF-2α(K429R)). As expected, Hu-VHL (Δ61+R161W) could catalyze the wild-type Hu-HIF-2α to form poly-ubiquitin chains, but not of the mutant, Hu-HIF-2α(K429R), under hypoxia (Figure 11C). In summary, BW-VHL could catalyze BW-HIF-2α to form poly-ubiquitin chains at K429, resulting in proteasomal degradation of BW-HIF-2α under hypoxia.

### BW-VHL enhances HIF-2α activity by modulating HIF-2α

**Figure 12:**
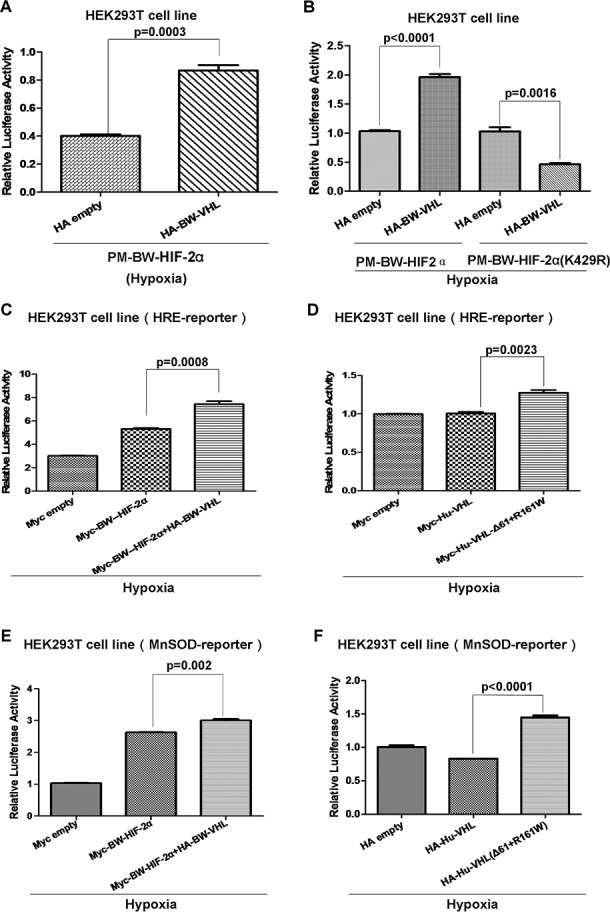
The beluga whale VHL enhances the beluga whale HIF-2α transcriptional activity under hypoxia **A**. The transcriptional activity of beluga whale HIF-2α was enhanced by beluga whale VHL overexpression under hypoxia. **B**. The beluga whale VHL overexpression suppressed the transcriptional activity of beluga whale HIF-2α mutant (K429R) compared to that of wide type BW-HIF-2α under hypoxia. **C**. The HRE reporter activity induced by beluga whale HIF-2α was enhanced by overexpression of the beluga whale VHL in HEK293T cells under hypoxia. **D**. The HRE reporter activity induced by hypoxia was enhanced by overexpression of human VHL mutant (Δ61+161R/W), while overexpression of wide type human VHL in HEK293T cells did not affect HRE promoter luciferase reporter activity under hypoxia condition. **E**. The MnSOD reporter activity induced by beluga whale HIF-2α was enhanced by overexpression of the beluga whale VHL in HEK293T cells under hypoxia. **F**. The MnSOD reporter activity induced by hypoxia was enhanced by overexpression of human VHL mutant (Δ61+161R/W), while overexpression of wide type human VHL in HEK293T cells did not affect MnSOD promoter luciferase reporter activity under hypoxia.

To determine the biological consequences of BW-VHL-mediated HIF-2α degradation under hypo-xia, we examined the effect of BW-VHL on HIF-2α transcriptional activity. We constructed artificial transcription factors by cloning BW-HIF-2α or BW-HIF-2α (K429R) into the PM vector (Clontech), which contains the GAL4 DNA binding domain(GAL4-DBD). A luciferase reporter pFR-Luc(Stratagene) harboring five-repeats of GAL4-DBD binding site in its promoter was used to monitor the transactivation activity of BW-HIF-2α or BW-HIF-2α (K429R) fused with GAL4-DBD. Luciferase assays in HEK293T cells confirmed that overexpression of BW-VHL enhanced the transcriptional activity of BW-HIF-2α, but not of BW-HIF-2α (K429R) mutant under hypoxia (Figure 12A and 12B). Furthermore, the HRE-reporter (which contains hypoxia response element) and MnSOD promoter luciferase reporter were used to validate this enhancement [43] and overexpression of BW-VHL enhanced activity of the HRE-reporter induced by BW-HIF-2α under hypoxia (Figure 12C). Interestingly, in HEK293T cells, overexpression of human wild-type VHL (Hu-VHL) did not change HER-reporter activity, but overexpression of human VHL(Δ61+R161W) mutant enhanced HRE-reporter activity under hypoxia, validating a unique feature owned of BW-VHL (Figure 12D). A MnSOD promoter luciferase reporter assays confirmed that BW-VHL and human VHL(Δ61+R161W) mutant enhanced MnSOD promoter reporter activity under hypoxia (Figure 12E and 12F).

**Figure 13:**
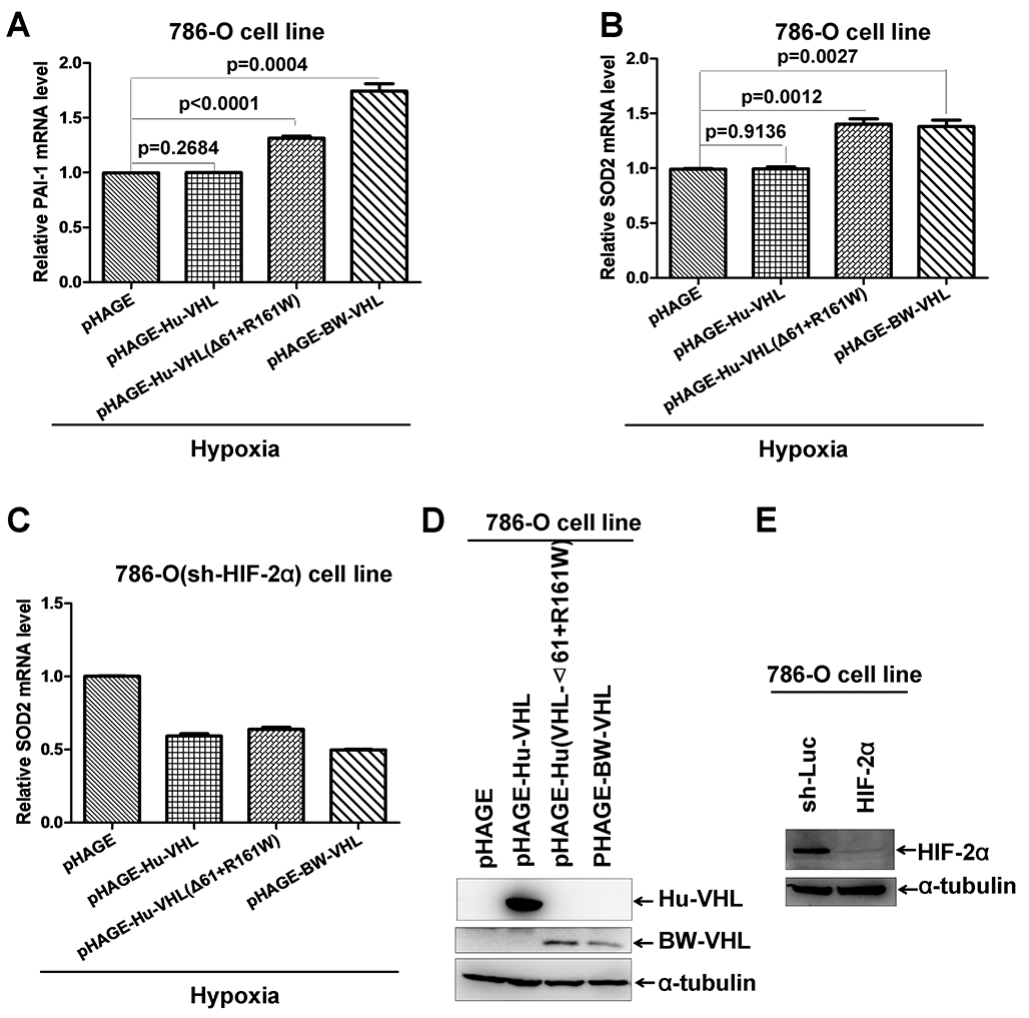
The beluga whale VHL enhances expression of HIF-2α down-stream genes **A**. Overexpression of the beluga whale VHL or human VHL mutant (Δ61+161R/W) in 786-O cells increased PAI-1 mRNA as revealed by semi-quantitative RT-RCR assays, but overexpression of human wildtype VHL did not do so. **B**. Overexpression of the beluga whale VHL or human VHL mutant (Δ61+161R/W) in 786-O cells increased SOD2 mRNA as revealed by semi-quantitative RT-RCR assays, but overexpression of human wildtype VHL did not do so. **C**. Knockdown of HIF-2α in 786-O cells abolished the enhancement of SOD2 expression by overexpression of the beluga whale VHL or human VHL mutant (Δ61+161R/W). **D**. Overexpression of human wildtype VHL, the beluga whale VHL and human VHL mutant (Δ61+161R/W) via lenti-virus infections was confirmed by Western blot analysis.

To determine whether the enhancement of BW-VHL on HIF-2α is mediated by endogenous HIF-2α, we used a human 780-O cell line, in which *Vhl* is deficient and only *HIF-2α* is presented [35, 44]. Overexpression of human wild-type VHL via lenti-virus infection in 780-O cells under hypoxia had no obviously effect on expression of *PAI-1* and *SOD2*, two well-defined HIF-2α down-stream genes (Figure 13A and 13B) [43]. However, overexpression of either human VHL(Δ61+R161W) mutant or BW-VHL via lenti-virus infection in 780-O cells under hypoxia enhanced expression of *PAI-1*and *SOD2* significantly (Figure 13A and 13B). Next, we knocked down of HIF-2α in 780-O cell via infection of lenti-virus expressing HIF-2α shRNA [45]. In this cell line, overexpression of wildtype human VHL, or human VHL(Δ61+R161W) mutant, or BW-VHL did not enhance *SOD* expression (Figure 13C). Overexpression of wild-type human VHL, human VHL(Δ61+R161W) mutant, and BW-VHL and knockdown of HIF-2α by shRNA were confirmed with Western blot assays (Figure 13D and 13E). In summary, BW-VHL can enhance HIF-2α down-stream gene expression via mediating HIF-2 degradation under hypoxia.

## DISCUSSION

Compared to the terrestrial mammals, cetaceans face frequent fluctuating oxygen concentrations [41] and as such have evolved unique adaptive capabilities [41]. As the dominant signaling governing cells in response to hypoxia, key factors in the hypoxia signaling, such as HIF-α and pVHL, should diverge between the terrestrial mammals and cetaceans. After aligning the amino acid sequences of HIF-α (HIF-1α and HIF-2α) and pVHL from different terrestrial mammals and different cetaceans respectively [40], HIF-α was found to be relatively evolutionarily conserved compared to pVHL, considering the protein primary structure. HIF-α subunits have similar domains and the key residues (proline residues), which are hydroxylated by PHDs. However, pVHL diverges at the N-terminus between cetaceans and the terrestrial mammals. BJ-VHL and FP-VHL lack the 53 amino acids corresponding to the N-terminus of pVHL in the terrestrial mammals; BW-VHL even lacks the 61 amino acids corresponding to the N-terminus of pVHL in terrestrial mammals. Also, SW-VHL is the longest one among the four cetaceans, but it lacks the 15 amino acids corresponding to the N-terminus of pVHL in terrestrial mammals. Although limited species of cetaceans and terrestrial mammals were chosen for amino acid sequence aligning assays of pVHL, differences in pVHL protein sequence between cetaceans and terrestrial mammals are obvious.

In humans, two proteins translated from one mRNA of the VHL gene have an alternate translation initial site: pVHL30 is the full length pVHL and pVHL19 is translated from the second methionine of pVHL mRNA which lacks the first 53 amino acids of the N-terminus [46, 47]. However, functional assays confirm no difference between pVHL30 and pVHL19, particularly for tumor suppression [48]. We report that pVHL from *L.vexillifer* (BJ-VHL) and *N*. *asiaeorientalis* (FP-VHL) lack the first 53 amino acids of the N-terminus, corresponds to human pVHL19. pVHL from *P. macrocephalus* lacks the first 61 amino acids of the N-terminus and this deletion in cetaceans suggests that the shorter form of pVHL has a different function than the longer form. Perhaps, the shorter pVHL is key to adapt to hypoxia. Clearly, whether the deletion in the N-terminus of pVHL conveys this adaptation warrants further study.

However, it seems that the 53 or 61 amino acid at the N-terminal of VHL is not required for BW-VHL to mediate HIF-2α degradation under hypoxia in that BJ-VHL and FP-VHL could not induce HIF-2α degradation under hypoxia although they lack the first 53 amino acids. Therefore, the difference between the C-terminus of pVHL might explain the divergence of hypoxic adaptation among cetaceans. We identified that W100 within BW-VHL is essential for BW-VHL's unique function. When W was mutated to R, the same as what occurs in BJ-VHL (R108), FP-VHL (R108) and Hu-VHL(R161), the BW-VHL(W100R) mutant lost the ability to induce HIF-2α degradation under hypoxia. Of note, W is a non-polar amino acid but R is a positively charged amino acid and this mutation may cause protein conformational changes due to electric charge alteration.

The unique feature of BW-VHL may contribute to the specific habitat choice of the beluga whale [49]. Frequent fluctuating of oxygen levels and low temperatures required adaptations [50] and the VHL might have gained particular features to distinguish it from that of other cetaceans during evolution.

As two master regulators of the hypoxia signaling pathway, HIF-1α and HIF-2α control similar gene expression programs in response to hypoxia [51], but the functional difference between HIF-1α and HIF-2α has been reported as well [29]. Human Tibetan PHD2 and HIF-2α underwent adaptive changes that influence hypoxia signaling and are considered necessary for Tibetan's high-altitude adaptation [27, 33, 52, 53]]. Here, we report that BW-VHL has features specific to HIF-2α, but not HIF-1α, suggesting a critical role of HIF-2α in hypoxic adaptation. Moreover, the main targeting site (K429) within HIF-2α by BW-VHL is located in the ODD domain of HIF-2a. We noticed that proline hydroxylation in the ODD domain is not required for BW-VHL-mediated HIF-2a degradation under hypoxia, so the ODD domain might be important for HIF-2a stability in addition to proline hydroxylation.

Degradation of HIF-2α by BW-VHL enhanced HIF-2α transactivity which was inconsistent with conventional views that BW-VHL targets HIF-2α for destruction and should inhibit transcriptional activity of HIF-2α. In fact, Ub ligases have been reported to act as transcriptional coactivators [54]. In addition, similar phenomenon has been reported for c-Myc regulation by Skp2. Skp2 mediates c-Myc degradation, but enhances c-Myc transcriptional activity [55]. Although mechanisms underlying these phenomena are still unknown, this kind of unusual regulation has been notified before. Here, we provide another example that E3 ligase can act as transcriptional coactivator as well as induce transcription factors for proteasomal degradation. To further reveal the mechanisms underlying this kind of phenomena will give insight into the relationship between the protein stability and the transactivity of transcriptional factors. Notably, when the degradation resistant mutant of HIF-2α (K429R) was co-transfected with BW-VHL, the enhancement of HIF-2α transactivity by BW-VHL under hypoxia was revised. It not only implicates that degradation of BW-HIF-2α by BW-VHL is required for the increase of HIF-2α transactivity, but also suggests that interaction between BW-HIF-2α (K429R) mutant and BW-VHL has some suppressive effects on BW-HIF-2α(K429R) transactivity when the degradation role of BW-VHL on BW-HIF-2α is abolished.

Intriguingly, as another master regulator of hypoxia signaling pathway, the protein level of BW-HIF-1α was increased instead of reducing by co-transfecting with BW-VHL under hypoxia (data not shown). In addition, BW-VHL could not mediate BW-HIF-1α degradation under normoxia (data not shown). Therefore, BW-VHL might have completely different effects on HIF-1α and HIF-2α, respectively, which needs to be further investigated.

Human cell lines were used for the assays [56] and these were validated for analysis of hypoxia signaling pathways found in cetaceans using human VHL mutant (hu-VHL (Δ61+R161W)) and human HIF-2α mutant (hu-HIF-2α(K429R)), which correspond to BW-VHL and BW-HIF-2α (K429R) respectively. Data from human VHL and HIF-2α mutants were consistent with those of BW-VHL and BW-HIF-2α. Thus, human cell lines can be used for studying cetaceans’ hypoxia signaling pathway, and suggest that hypoxia signaling is evolutionarily conserved across cetaceans to human.

The cetacean species studied were limited but unique features were identified, particularly for VHL of the beluga whale. Using more cetaceans in future studies will increase our understanding of factors in hypoxia signaling pathway as well as help us better understand how cetaceans adapt to and tolerate hypoxia.

## MATERIALS AND METHODS

### Sample collections

Kidney samples were collected from male Yangtze finless porpoise (*Neophocaena asiaeorientalis asiaeorietalis*) caught in fishing nets in the Honghu section of the Yangtze River, which died 2 weeks after rescue. A blood sample was obtained from a male beluga whale (*Delphinapterus leucas*) at Wuhan Polar Ocean World. Necropsy and sampling were performed in accordance with all ethical guidelines and legal requirements in China. The protocols for sampling were approved by the research committee of Institute of Hydrobiology, Chinese Academy of Sciences.

### Molecular cloning and sequence analysis

Beluga whale DNA was extracted from blood using a QIAamp DNA Blood Mini Kit (Qiagen, Hilden, Germany). The Yangtze finless porpoise total RNA was extracted from kidney tissue using TRIzol (Invitrogen, Karlsruhe, Germany). Beluga whale RNA was extracted from blood using a QIAamp RNA Blood Mini Kit (Qiagen, Hilden, Germany) according to the manufacturer's protocol. Primers for Yangtze finless porpoise HIF-2α were designed using Primer Premier Version 5.00 (Premier Biosoft International, Palo Alto, California) based on conserved sequences of HIF-2α 5′UTR and 3′UTR in the killer whale (*Orcinus orca*), minke whale(*Balaenoptera acutorostrata*), cattle(*Bos tarurs*), and dolphin (*Tursiops truncatus*) genomes. Because HIF-2α could not be detected in peripheral blood leukocytes, primers for beluga whale HIF-2α were designed based on conserved sequences of HIF-2α exons in cetacean genomes. Primers for amplifying beluga whale HIF2α are listed in Supplementary Table 1. Primers for Yangtze finless porpoise and beluga whale VHL were designed based on conserved sequences of known cetaceans VHL 5′UTR and exon 3. cDNA sequences of HIF-2α and VHL of the baiji (*Lipotes vexillifer*, BJ) and sperm whale (*Physeter macrocephalus*, SW) were predicted based the genomic sequences (BJ, HIF-2α: Accession number XM_007459966.1, VHL: XM_007113722.1; SW, HIF-2α: XM_007105117.1, VHL: XM_007113722.1). Primers for amplifying Yangtze finless porpoise (FP) HIF-2α were :5′-CCACAGCGAAGGTAGCGG-3′ (forward) and 5′-AAGGGGGTGCCTGTCAGTG-3′ (reverse). Primers for amplifying Yangtze finless porpoise and beluga whale (BW) VHL were: 5′-AGTTCTGGCTCCGGGAGGTA-3′ (forward) and 5′-TCAATTAAAATTTTCAGTCTCC’ (reverse).

For phylogenic analysis, the sequences of known HIF-2α and VHL were obtained from GenBank (http://www.ncbi.nlm.nih.gov/genbank/) or Ensembl (http://www.ensembl.org) database. Tree searches were made using Bayesian analysis in MrBayes 3.1.2 [57].

### Plasmid constructs

Full-length HIF-2α and VHL cDNAs from Yangtze finless porpoise were generated using RT-PCR with primers that incorporated restriction sites and were then inserted into expression vector. Beluga whale VHL was cloned from cDNA and inserted into pCMV-HA expression vector. The full-length HIF-2α and VHL of sperm whale and the baiji, as well as HIF-2α from beluga whale were synthesized by Wuhan Tianyi Huiyuan Bioscience & Technology Corporation (Wuhan, China).

### Antibodies and reagents

Antibodies used were as follows: anti-c-Myc antibody (9E10, Santa Cruz; A0309, ABclonal; D84C12, Cell Signaling), anti-HA antibody (Covance) and anti-α-tubulin antibody (EPR1333, Epitomics). The reagents used were as follows: MG132 (Calbiochem), cycloheximide (Sigma-Aldrich).

### *In vivo* ubiquitination assays

HEK293T cells were co-transfected with the indicated plasmids by Vigofect (Vigorous Biotech., Beijing). After transfection for 24h, the cells were collected, lysed and subjected to immunoprecipitation by Ni2^+^- NTA beads (Novagen), then examined by Western blotting analysis using anti-Myc antibody. The ubiquitin mutants have been described previously [58].

### Luciferase reporter assays

Cells were grown in 24-well plates and transfected with the indicated amounts of plasmids using VigoFect, as well as with pTK-Renilla used as an internal control. Transfected cells were incubated in fresh medium under normoxia (21 % O2) for 6 h and were then exposed to hypoxia (1 % O2) for 18–20 h. Luciferase activity in the cell lysates was determined using a Dual-luciferase reporter assay system (Promega).

### Western blot analysis

No suitable whale cell lines were available, thus human embryonic kidney 293T (HEK293T) cell line was used for the experiments [56]. HEK293T cells were cultured in Dulbecco's modified Eagle's medium (DMEM) supplemented with 10 % fetal bovine serum (HyClone, Logan, UT, USA).

For protein expression assays, we transiently transfected HEK293T cells with the indicated plasmids using VigoFect reagent. For hypoxic treatments, the cells were transfected for 6 hr, and then treated for 18-20 hr in a hypoxia (1 % O2) chamber (Ruskinn INVIVO_2_400). For re-oxygenation, the cells were treated under hypoxia (1 % O2) for 18 hr, and then put into the regular incubator (21% O2) for the indicated times.

Cells were harvested in lysis buffer at the indicated times. After the transfected cells were incubated in hypoxic or normoxic conditions for 18–20 h, they were lysed in radio immunoprecipitation assay (RIPA) buffer (50 mM TrispH 7.4, 150 mM NaCl, 1 mM EDTA, 1 % Nonidet P-40,0.5 % sodium deoxycholine, and 1 mM NaF) that was supplemented with 1 mM PMSF, 1 mM Na3VO4, and 1 mM proteinase inhibitor immediately before use. α-tubulin was used as a loading control. A Fujifilm LAS4000 mini luminescent image analyzer was used to photograph the blots, and the density of the protein bands was quantified using Multi Gauge V3.0 analyzer software (FujiFilm Corp., Tokyo, Japan).

### Lentivirus-mediated gene overexpression and knockdown

Human VHL, Human VHL(Δ61+R161W) and Beluga whale VHL were subcloned into the lentivirus vector pHAGE-CMV-MCS-IZsGreen. Lentiviruses for gene overexpression were generated by transfecting HEK293T cells with a transducing vector, and the packaging vectors, psPAX2 and pMD2.G. After transfection for 8 h, the medium was replaced with fresh DMEM medium with 10% FBS. After 40 h, virus particles in the medium were harvested, filtered, and transduced into 786-O cells. Polybrene (8 μg/mL) was added to the medium to improve infection efficiency. Lentivirus-mediated Knockdown of HIF-2α in 786-O cells have been described previously [50].

### Semi-quantitative real-time RT-PCR

Total RNA was extracted from cells by using TRIzol reagent (Invitrogen), and cDNA was synthesized using a first-strand cDNA synthesis kit (Fermentas) following the manufacturer's instructions. Human PAI-1,SOD2 cDNA were amplified with the following primer sequence : PAI-1 forward, 5′-AAAGGCAACATGACCAGGCT-3′, and reverse, 5′-GGGAGAACTTGGGCAGAACC-3′; SOD2 forward, 5′-AGCATGTTGAGCCGGGCAGT-3′, and reverse, 5′-AGGTTGTTCACGTAGGCCGC-3′; 18S rRNA was used as an internal control. The primers for 18S rRNA were: 5′-TCAACTTCGATGGTAGTCGCCGT-3′, and 5′-TCCTTGGATGTGGTAGCCGTTCT-3′

### Statistical analysis

Luciferase and RT-PCR assay data are reported as mean ± S.E.M. of three independent experiments performed in triplicate. The statistical analysis was performed using GraphPad Prism 5 (unpaired t-test) (GraphPad Software Inc.).

## SUPPLEMENTARY MATERIALS FIGURES AND TABLES


