# Supplementary material for: Beluga whale pVHL enhances HIF-2α activity via inducing HIF-2α proteasomal degradation under hypoxia
Source: Oncotarget. 2017 Feb 2;8(26):42272–87. doi: 10.18632/oncotarget.15038 (PMC5522066; doi:10.18632/oncotarget.15038)
Supplement: Supplementary file 1 [file oncotarget-08-42272-s001.pdf]

Beluga whale pVHL enhances HIF-2α activity via inducing HIF-2α proteasomal degradation under hypoxia

SUPPLEMENTARY MATERIALS

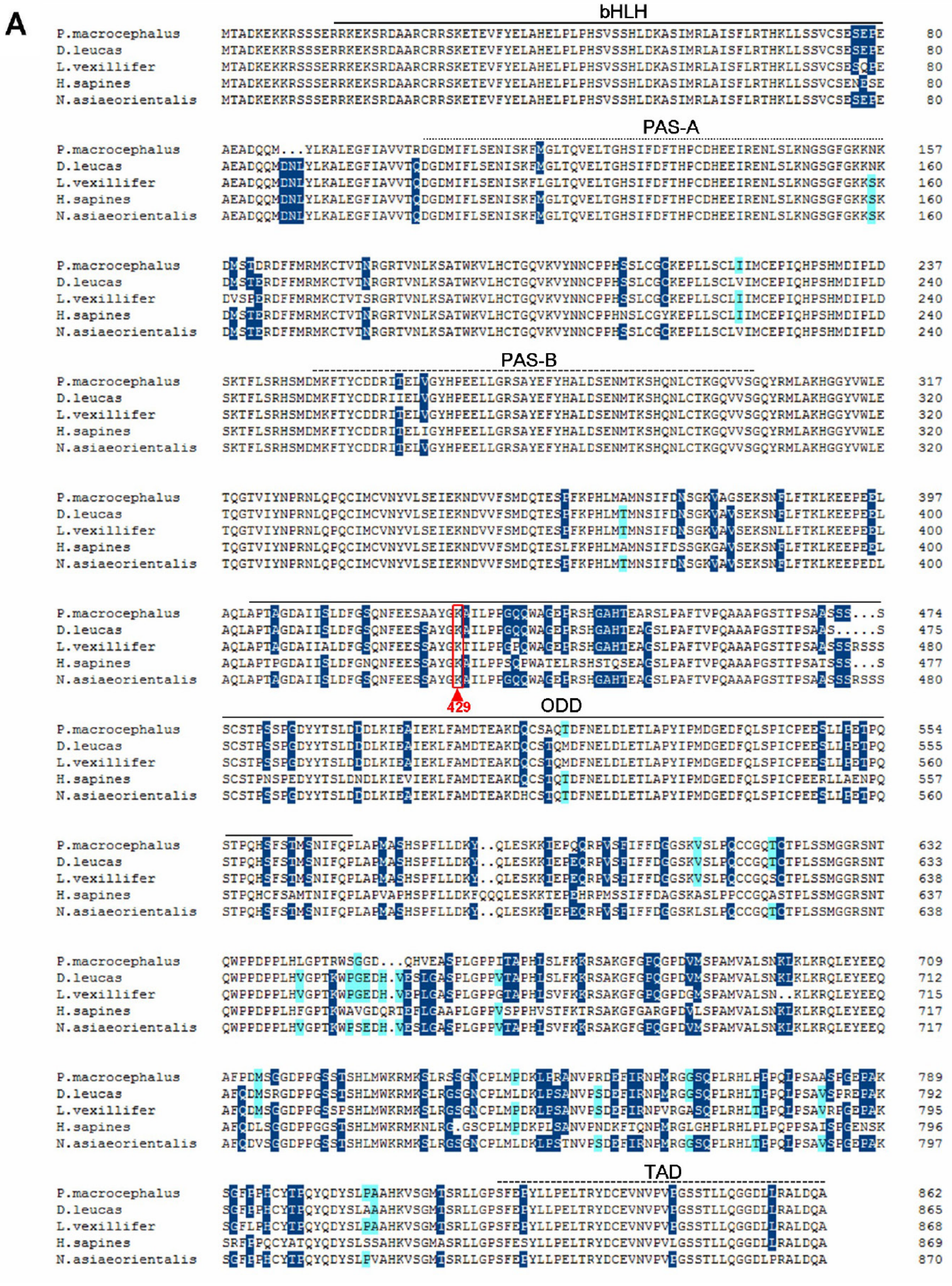

Supplementary Figure 1: A. Sequence alignment of HIF-2α from the baiji (*Lipotes vexillifer*, Accession number XM_007459966.1), the Yangtze finless porpoise (*Neophocaena asiaeorientalis asiaeorientalis*, KX227380), the beluga whale (*Delphinapterus leucas*, KX227381), the sperm whale (*Physeter macrocephalus*, XM_007105117.1) and human (*Homo sapiens*, U81984.1). (*Continued*)

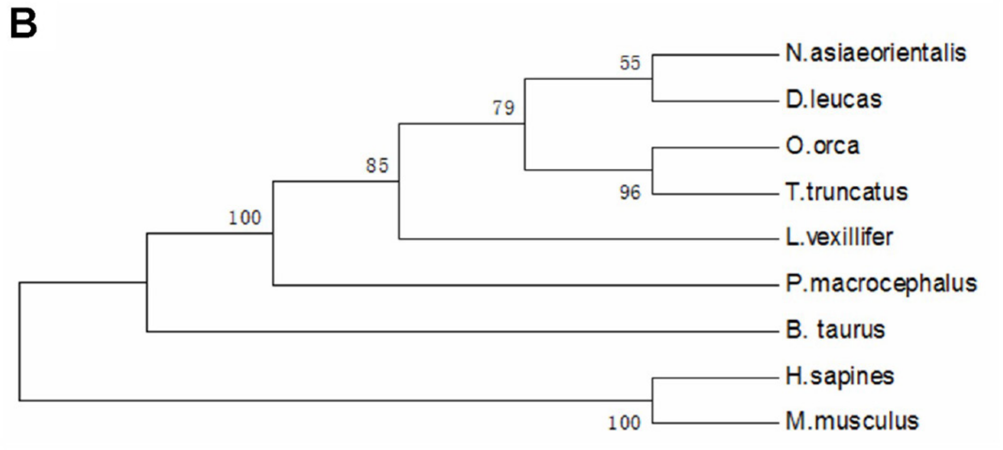

Supplementary Figure 1: (*Continued*) B. Phylogenetic tree based on the amino acid sequences of HIF-2α by Bayesian analysis.

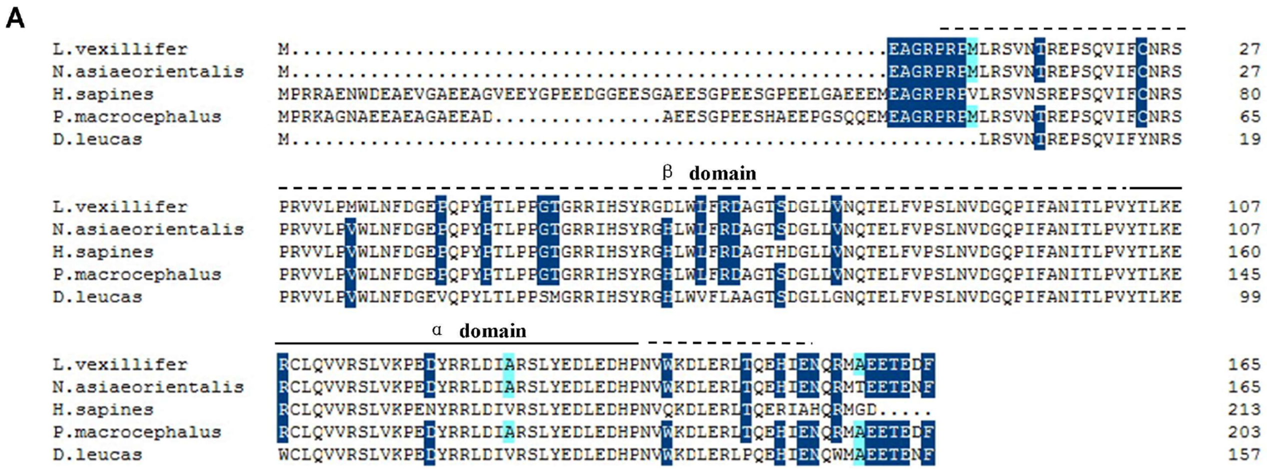

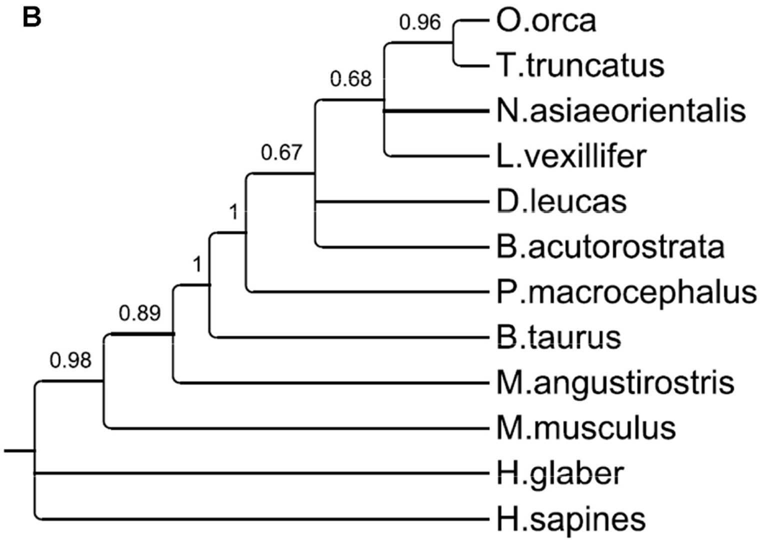

Supplemental Figure 2: A. Sequence alignment of VHL from the baiji (Accession number XM_007472386.1), the Yangtze finless porpoise (KX227382), the beluga whale (KX227383), the sperm whale (XM_007113722.1) and human (NM_000551.3). **B.** Phylogenetic tree based on the amino acid sequences of VHL by Bayesian analysis.

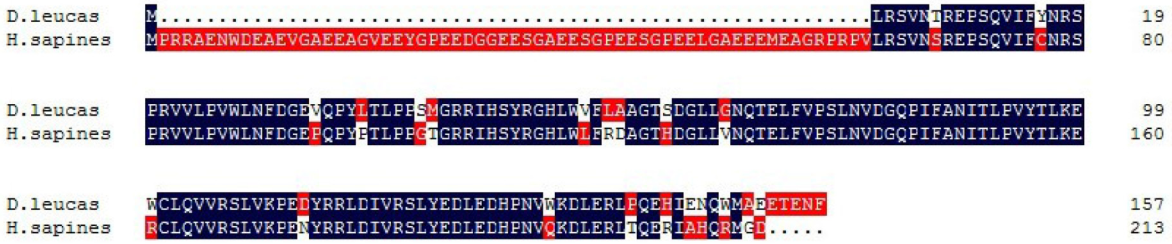

Supplemental Figure 3: Sequence alignment of the beluga whale VHL and human pVHL. The sites that differ between beluga whale VHL and human pVHL are indicated by red color.

Supplementary Table 1: The PCR primers used to amplify beluga whale HIF-2α

Primers	Sequence (5′ to 3′)	Position	Product (bp)
F1	AGTGAAGGCATCGCGGTGAC	5’ UTR	80
R1	CTGGGGGAGTGGGCTTACTTTT	Intron 1	
F2	AGGGGTGTGATGGGATGA	Exon 2	191
R2	CCACCGCTGGAAAGATGT	Exon 2	
F3+4	GTGACCCCTACTGCTGC	Exon 3	650
R3+4	CCTAACCTGAAGGAGTGC	Exon 4	
F5+6	CCCCCTCTGAATACAAAC	Exon 5	416
R5+6	CACCCAGCAATACAACAT	Exon 6	
F7	AATCAYAGAACTGRTTG	Exon 7	107
R7	AGTTCTGGTGRCTYTTG	Exon 7	
F8+9	TGTGCACCAAGGGTCAG	Exon 8	1014
R8+9	CGAAATCCAGAGMRATGATG	Exon 9	
F10+11	GGAVCCMGAACTTTGAGGAGTC	Exon 10	861
R10+11	CTGGGYGCYRCACTGGT	Exon 11	
F12	ACKGACTTYAAYGAGCT	Exon 12	476
R12	CTCTTCTTGARCAHGGMGA	Exon 12	
F13+14+15	GTCTGCMAAGGGYTTYGG	Exon 13	769
R13+14+15	CTGACAYCTTGTGRGCCG	Exon 15	
F16	GTCTGCMAAGGGYTTYGG	Exon 16	152
R16	CTGACAYCTTGTGRGCCG	Exon 16	
